# Interface-Induced Magnetization Reversal and Multistate Magnetoresistance in Fe_3_GaTe_2_/Ni_80_Fe_20_ Heterostructure

**DOI:** 10.3390/nano16201304

**Published:** 2026-10-15

**Authors:** Guangcheng Wang, Lin Ma, Shuo Xu, Jihui Li, Meiyin Yang, Zijing Zhao, Xiaolei Wang

**Affiliations:** 1 School of Physics and Optoelectronic Engineering, Beijing University of Technology, Beijing 100124, China; 2 Key Laboratory of Advanced Functional Materials, Ministry of Education, College of Materials Science and Engineering, Beijing University of Technology, Beijing 100124, China; 3 Key Laboratory of Fabrication Technologies for Integrated Circuits, Chinese Academy of Sciences, Beijing 100029, China; 4 Institute of Microelectronics of the Chinese Academy of Sciences, Beijing 100029, China

**Keywords:** Fe_3_GaTe_2_, Permalloy, mixed-dimensional heterostructures, interfacial magnetic pinning, multistate magnetoresistance

## Abstract

Mixed-dimensional heterostructures integrating two-dimensional (2D) van der Waals magnets with conventional ferromagnetic films unify the versatile functionalities of both material classes and offer enormous potential for magnetic sensing, non-volatile data storage, and reconfigurable spintronic devices. More importantly, 2D domain evolution can modulate the magnetization reversal and magnetotransport of the adjacent ferromagnetic layer through interface engineering. Here, we construct a vertically stacked Fe_3_GaTe_2_/Ni_80_Fe_20_ (Permalloy, Py) heterostructure. Magnetometry measurements confirm the magnetic anisotropies of both constituents and labyrinthine domains in Fe_3_GaTe_2_. Compared with the narrow, one-step low-field anisotropic magnetoresistance features of isolated Py, the heterostructure exhibits multipeak resistance responses and pronounced hysteresis. Asymmetric minor-loop field-sweeping measurements further reveal a strong dependence on the positive maximum field, which modifies the loop morphology and produces distinct remanent resistance states at zero field. These observations are consistent with a domain-mediated interfacial pinning mechanism, in which Fe_3_GaTe_2_ domains and domain walls modify the interfacial magnetic environment, thereby pinning adjacent Py moments and promoting their progressive, multistep reversal. These findings provide a route toward multistate spintronic devices based on mixed-dimensional magnetic heterostructures.

## 1. Introduction

Interfacial interactions in magnetic heterostructures provide an effective pathway to modulate magnetic anisotropy, magnetization reversals, and magnetotransport behavior, rendering such systems promising platforms for magnetic sensing, nonvolatile information storage, and reconfigurable spintronic devices [1,2,3,4,5,6]. Conventional magnetic devices generally rely on the relative magnetization orientations of uniform ferromagnetic films and therefore exhibit predominantly binary resistance responses [7,8]. In contrast, two-dimensional (2D) magnets host rich domain distributions and domain-wall configurations, and their magnetic states depend not only on the instantaneous applied field but also on the preceding field-sweep history [9,10]. Related studies of one-dimensional magnetic nanochains and nanowires have demonstrated the role of local geometry and magnetic anisotropy in domain formation and magnetization reversal, alongside magnetoresistive functionality in nanochain devices [11]. Through interfacial interactions, such field-dependent domain evolution can reconfigure the magnetization reversal of the adjacent magnetic layer, thereby generating multilevel remanent resistance states beyond the binary response of uniform magnetic films [12,13,14] and providing a promising route toward multistate and programmable magnetotransport.

Permalloy [Ni_80_Fe_20_ (Py)] is a typical soft ferromagnetic material with low coercivity and an in-plane (IP) magnetic easy axis that arises from the shape anisotropy of the thin film [15,16]. Because anisotropic magnetoresistance (AMR) depends on the angle between the magnetization and current directions, the out-of-plane (OOP) canting and nonuniform reversal of the Py magnetization can be electrically detected through its magnetoresistance measurements [17,18]. Moreover, the low coercivity of Py makes its magnetization nucleation, domain-wall depinning, and reversal highly sensitive to local effective fields and interfacial pinning barriers [17,19]. Py can therefore serve both as a magnetic layer responding to interfacial interactions and as an electrical probe of the magnetic-state evolution in an adjacent magnet. In an isolated Py film, however, magnetization reversal is mainly governed by shape anisotropy, defects, and device geometry, resulting in a magnetoresistance response confined to a narrow low-field range and hindering the realization of diverse tunable states.

2D van der Waals ferromagnets provide a new material basis for engineering magnetic interfaces. Their dangling-bond-free surfaces and relaxed lattice-matching requirements facilitate mixed-dimensional integration with conventional ferromagnetic films [2,3,20,21]. Among them, metallic Fe_3_GaTe_2_ (FGaT) has attracted considerable attention because it retains ferromagnetism at room temperature and exhibits strong perpendicular magnetic anisotropy and a pronounced anomalous Hall response [22,23,24]. More importantly, FGaT hosts labyrinthine domains comprising regions with opposite OOP magnetizations and domain walls [25,26]. The interfacial magnetic environment established by the FGaT domains may locally cant or pin the interfacial moments, thereby modifying local switching fields and overall reversal pathway [27,28,29]. Magnetic-layer configurations and nanoscale structural design provide established routes to complex magnetization reversal and multistate magnetotransport [30]. A complementary strategy is to combine a perpendicular multidomain magnet with an in-plane soft ferromagnet, allowing field-history-dependent domain configurations to potentially influence the adjacent layer through interfacial interactions. The FGaT/Py heterostructure provides a platform for investigating this possibility and its relationship to electrically distinguishable remanent states.

Here, we construct a mixed-dimensional magnetic heterostructure comprising an upper two-dimensional FGaT layer and a lower IP soft-magnetic Py film. Magnetic measurements establish the OOP and IP magnetic easy axes of FGaT and Py, respectively, while magnetic force microscopy and magneto-optical Kerr microscopy reveal labyrinthine domains in FGaT. By comparing the angle-dependent transport responses of isolated FGaT, isolated Py, and the FGaT/Py heterostructure, we find that the heterostructure exhibits markedly broadened multipeak magnetoresistance features and asymmetric field-sweep loops. Asymmetric minor-loop measurements further show that varying the positive turning field *H*_max_ modifies the loop morphology and produces distinct remanent resistance states at zero field. Based on independent magnetic-domain imaging and measurements of the devices, we propose a domain-mediated interfacial pinning mechanism: the FGaT domain configuration modifies the interfacial pinning barriers experienced by Py, thereby regulating its magnetization nucleation, depinning, and multistep reversal. These findings suggest that perpendicular multidomain two-dimensional magnets can serve as active interfacial control layers for reconfiguring conventional IP soft ferromagnets, providing an interface-engineering route toward mixed-dimensional multistate and reconfigurable spintronic devices.

## 2. Methods

### 2.1. Sample and Device Preparation

The bulk FGaT was fabricated using the self-flux method. High-purity Fe powder (99.99%), Ga lumps (99.99%) and Te powder (99.999%) (Aladdin Co., Shanghai, China) were mixed at atomic ratios of 1:1:2 in a sealed quartz tube with high vacuum, in which the Ga and Te also acted as the flux. The quartz tubes were placed in a muffle furnace and heated to 1273 K in 1 h. After holding for 24 h, the temperature was rapidly lowered to 1153 K in 1 h. Then, the temperature was slowly lowered to 1053 K over a period of 120 h. Finally, single crystal of Fe_3_GaTe_2_ was obtained by centrifugation. Thin FGaT flakes were obtained from the bulk crystals by mechanical exfoliation. The Pys (30 nm) were prepatterned onto a SiO_2_ (300 nm)/Si substrate using magnetron sputtering. The base pressure was 6 × 10^−6^ Pa, and the Ar gas pressure during growth was 0.6 Pa. An exfoliated FGaT flake was then aligned and placed onto the Py channel using a dry-transfer technique, forming a vertically stacked FGaT/Py heterostructure. The Py device was fabricated using a photolithography-assisted lift-off process. Briefly, the device pattern was defined on the substrate by ultraviolet photolithography, followed by Py deposition and lift-off to form the patterned Py channel. The FGaT flake used for MFM and MOKE imaging was approximately 156 nm thick, whereas the flake used in the transport device was approximately 123 nm thick, as determined by atomic force microscopy (AFM; Figure S1).

### 2.2. Measurement

The crystal structures of FGaT and Py were examined by X-ray diffraction (XRD) using a Bruker D8 Advance diffractometer (Bruker AXS, Karlsruhe, Germany) with Cu Kα radiation (λ = 1.5406Å). The Raman spectra were measured using the Witec Confocal Raman spectroscope (WITec, Ulm, Germany) with a 532 nm wavelength excitation laser and 1 μm spot size. The magnetic domain measurements were performed with an atomic force microscope (Asylum Research, Santa Barbara, CA, USA). The domain images of film stacks were measured by the magneto-optical Kerr effect (MOKE) (Truth Instruments, Qingdao, China). The *M-T* (magnetization vs. temperature) and *M-H* (magnetization vs. field) curves of FGaT and Py were characterized by Vibrating Sample Magnetometer (VSM). Four-probe electrical measurements of electrical transport dependent on magnetic field were conducted using electromagnet (EM3, East Changing, Beijing, China) with a fixed 0.1 mA DC current applied to the channel.

## 3. Results and Discussion

**Figure 1:**
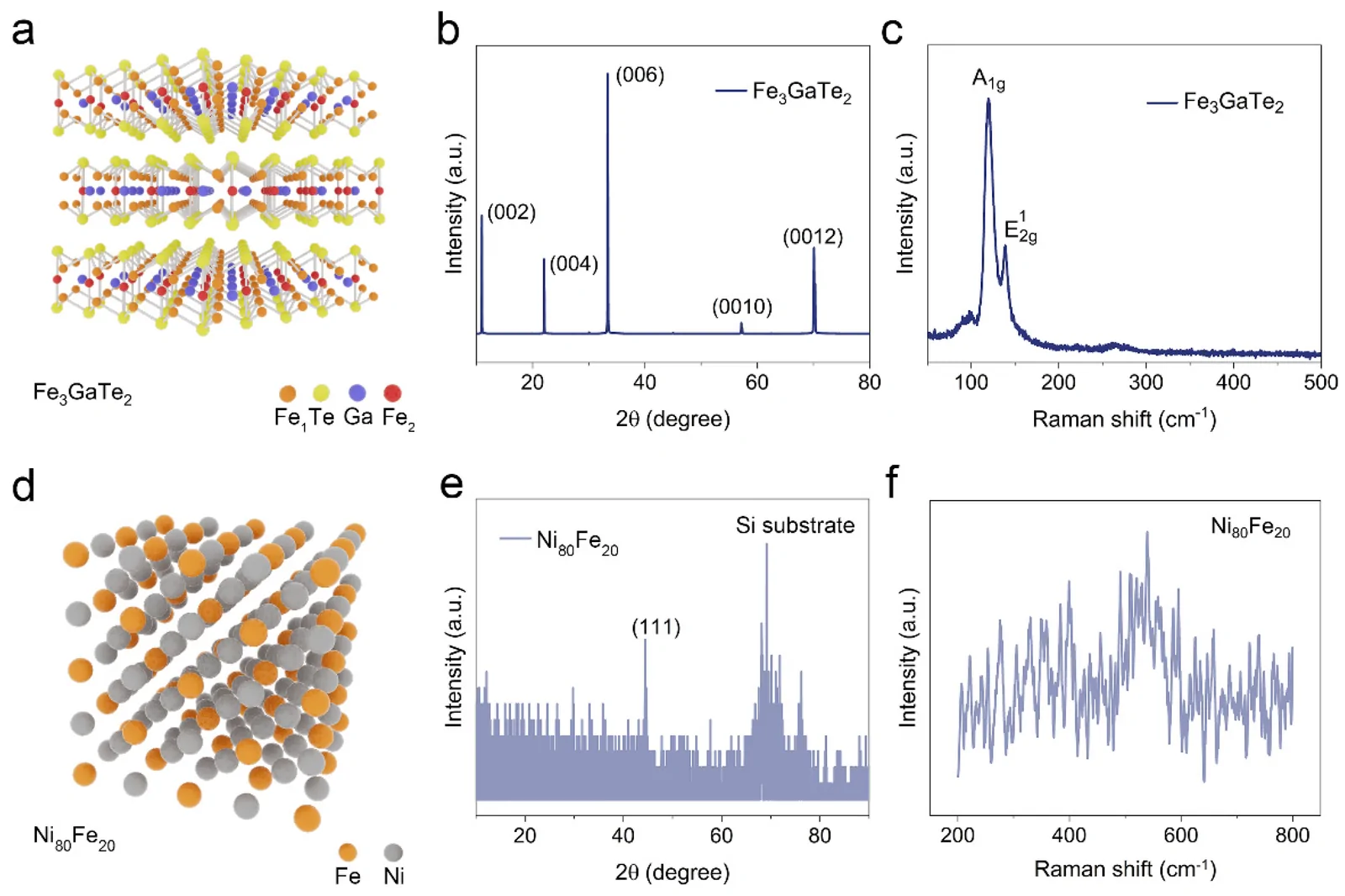
Structural characterization of Fe_3_GaTe_2_ and Ni_80_Fe_20_ Permalloy (Py). (**a**) Structural model of the layered crystal structure of Fe_3_GaTe_2_. (**b**) X-ray diffraction pattern of the Fe_3_GaTe_2_ crystal, showing a series of (00l) reflections. (**c**) Raman spectrum of Fe_3_GaTe_2_ flake with the characteristic (A_1g_) and (E_2g_^1^) vibrational modes. (**d**) Structural model of the chemically disordered face-centered-cubic Ni_80_Fe_20_ alloy. (**e**) X-ray diffraction pattern of the Py film. (**f**) Raman spectrum of the Py film.

Figure 1 summarizes the structural characterization of the Fe_3_GaTe_2_ (FGaT) crystal and the Ni_80_Fe_20_ Permalloy (Py) film used to construct the heterostructure. As illustrated in Figure 1a, Fe_3_GaTe_2_ possesses a layered van der Waals structure, in which the Fe_3_Ga slabs are sandwiched between Te layers and stacked along the crystallographic *c* axis [22,31]. XRD pattern in Figure 1b is dominated by a series of (00l) reflections, confirming the layered crystalline structure and preferential *c*-axis orientation of FGaT. The characteristic Raman modes shown in Figure 1c further support the structural identity of the Fe_3_GaTe_2_ crystal [32]. Py can be described as a chemically disordered face-centered-cubic solid solution, in which Ni and Fe atoms statistically occupy the fcc lattice sites (Figure 1d). The XRD pattern of the Py film exhibits a peak assigned to the (111) plane, together with the dominant diffraction signals from the substrate (Figure 1e) [33,34]. The Raman spectrum of the Py film shows a broad feature around 500–580 cm^−1^ without well-resolved vibrational peaks. Similar broad spectral signatures in this wavenumber range have also been observed in NiFe-based samples. Nevertheless, the present spectrum serves as supplementary characterization rather than conclusive evidence for the identification of metallic Py. These structural results confirm the high quality of the fabricated materials, enabling further exploration of interfacial magnetic coupling between layered FGaT and Py thin film.

**Figure 2:**
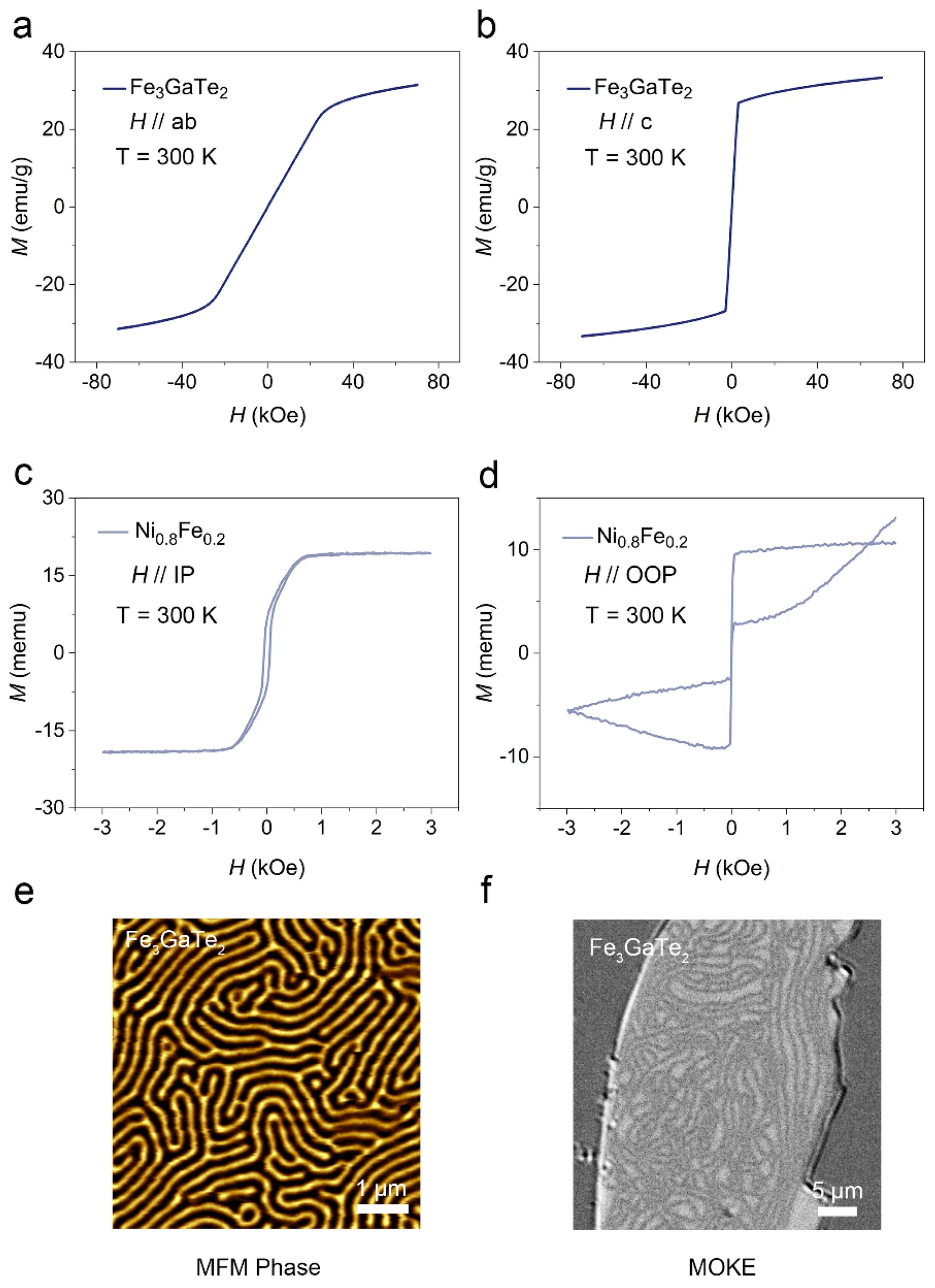
Magnetic anisotropies and multidomain characteristics of Fe_3_GaTe_2_ and Py. (**a**,**b**) Magnetic hysteresis loops of Fe_3_GaTe_2_ measured at 300 K with the magnetic field applied parallel to the *ab* plane (**a**) and along the crystallographic *c* axis (**b**). (**c**,**d**) Magnetic hysteresis loops of the Py film measured with the magnetic field applied IP (**c**) and OOP (**d**). (**e**) MFM phase image showing the labyrinthine magnetic-domain configuration of Fe_3_GaTe_2_. (**f**) MOKE image showing large-area labyrinthine domains in Fe_3_GaTe_2_. MOKE image was acquired at an applied field of −100 mT during the field sweep.

Figure 2 compares the magnetic properties of FGaT and Py. Figure 2a presents the in-plane magnetic hysteresis loop of FGaT at 300 K [12]. The gradual increase in magnetization over a broad field range indicates that the *ab* plane corresponds to the magnetically hard direction. In contrast, the out-of-plane (OOP) *M–H* loop measured with (*H*//c) exhibits a pronounced hysteresis and rapid magnetization reversal at relatively low fields, confirming that the magnetic easy axis of FGaT is along the OOP direction. The OOP MOKE loop of ultrathin FGaT exhibits a nearly rectangular profile with abrupt switching and a finite remanent Kerr signal (Figure S2). This optical measurement complements the magnetometry results and further supports the perpendicular magnetic easy axis of FGaT. In contrast, the in-plane (IP) *M–H* loop of the Py film shows a small coercive field and a low saturation field, demonstrating its characteristic soft-ferromagnetic behavior with an IP magnetic easy axis. When the magnetic field is applied perpendicular to the film plane, the Py magnetization evolves gradually over a considerably larger field range. This hard-axis-like response originates primarily from the shape anisotropy of the thin film and indicates a field-induced canting of the Py magnetization from the IP direction toward the OOP direction. Therefore, the magnetic hysteresis loops measured at 300 K demonstrate that FGaT possesses an OOP magnetic easy axis, whereas the Py film exhibits characteristic IP soft-ferromagnetic behavior. In addition, the MFM phase image in Figure 2e, acquired at zero applied field after zero-field cooling, reveals a local labyrinthine magnetic domain pattern in FGaT. Consistently, the MOKE image in Figure 2f displays alternating bright and dark labyrinthine contrasts over a larger field of view, corresponding to domains with opposite OOP magnetization components. Both images were acquired from the same characterization flake. These complementary magnetic-imaging results confirm that FGaT supports perpendicular multidomain configurations.

**Figure 3:**
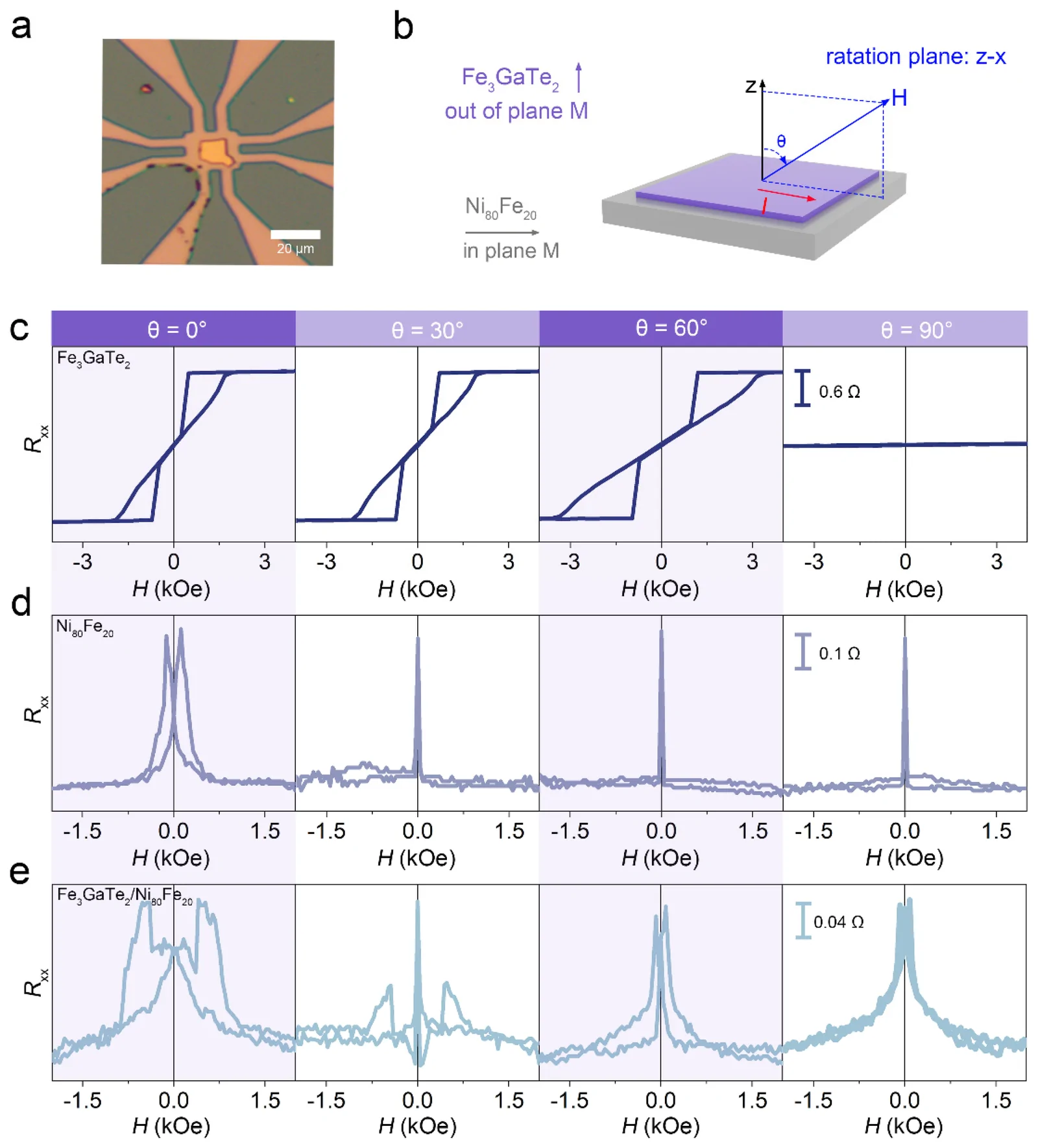
Angle-dependent magnetotransport of FGaT, Py, and the FGaT/Py heterostructure. (**a**) Optical micrograph of the FGaT/Py heterostructure device. (**b**) Schematic of the device geometry and magnetic-field rotation. The current is applied along the x direction, and *θ* is defined as the angle between the magnetic field and the OOP *z*-axis. Thus, *θ* = 0° and 90° correspond to OOP and IP field configurations, respectively. (**c**–**e**) Longitudinal resistance as a function of magnetic field at *θ* = 0°, 30°, 60°, and 90° for FGaT (**c**), Py (**d**), and the FGaT/Py heterostructure (**e**).

Figure 3 compares the angle-dependent longitudinal magnetoresistance of thick FGaT, Py, and the FGaT/Py heterostructure. The magnetic field was rotated in the *x*–*z* plane, with *θ* defined as the angle between the field and the OOP *z*-axis. Although the transport curves of FGaT were performed using a Hall-bar device geometry, the intrinsic longitudinal resistance (*R*_xx_) is obscured by the substantially larger anomalous Hall signal. A slight transverse misalignment of the voltage contacts introduces an anomalous Hall contribution into the measured longitudinal resistance. Therefore, the hysteretic signal in Figure 3c is dominated by anomalous Hall admixture and is used primarily as an electrical indicator of the OOP magnetization reversal of FGaT. The nominal longitudinal resistance of the heterostructure may contain a small anomalous Hall contribution from FGaT, yet this does not invalidate the overall experimental observations. The broadened multipeak response establishes an electrical behavior distinct from that of isolated Py. Domain-mediated interfacial modulation is therefore considered a plausible interpretation, rather than a unique assignment of all resistance features to Py magnetization reversal.

The hysteresis is progressively suppressed and the coercivity increases as the field is rotated toward the IP plane, consistent with its perpendicular magnetic anisotropy and the decreasing OOP field component. An independent transverse-resistance measurement reveals a pronounced hysteretic Hall response in thick FGaT nanoflakes under an OOP field (Figure S3), providing an electrical signature of its perpendicular magnetization reversal. This observation supports the interpretation that an anomalous Hall contribution can enter the nominal longitudinal voltage through contact misalignment and contribute to the hysteretic response in Figure 3c. In Figure 3d, although Py has an IP magnetic easy axis, two small resistance peaks are observed in the nominally OOP field configuration. These peaks do not indicate intrinsic OOP switching of Py but arise from a small angular misalignment between the applied field and the film normal. Even a misalignment of approximately 1° generates an in-plane field component sufficient to reverse the soft Py magnetization [16,35]. As the field is rotated toward the film plane, the increasing in-plane component reduces the field required to reverse the Py magnetization. Consequently, the two misalignment-induced AMR peaks progressively shift toward zero field and evolve into a sharp low-field feature in the IP configuration.

**Figure 4:**
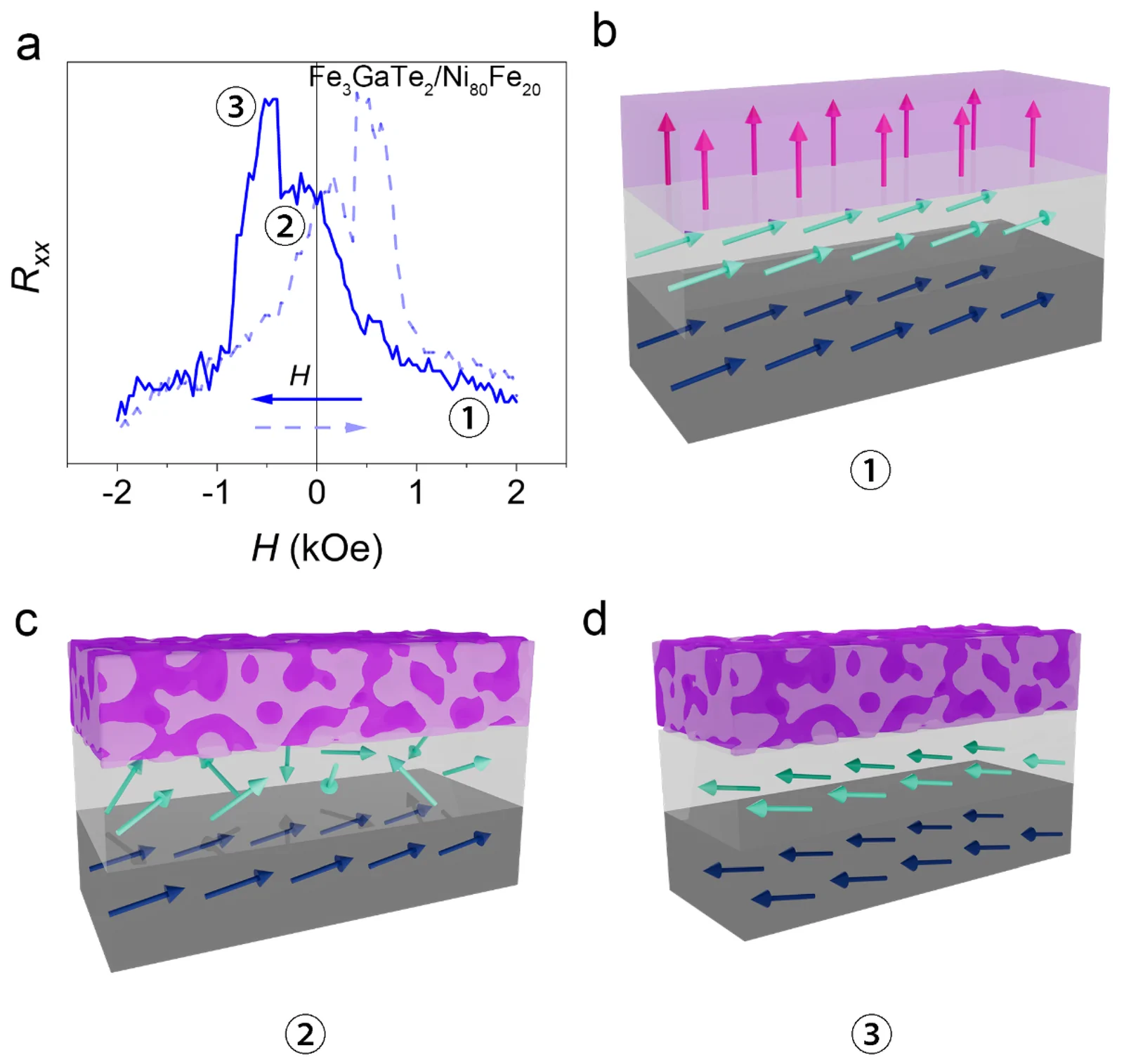
Proposed domain-mediated interfacial pinning scenario in the FGaT/Py heterostructure. (**a**) Longitudinal resistance of the heterostructure under an OOP magnetic field, with three representative resistance states. The solid and dashed curves in figure (**a**) represent the decreasing- and increasing-field sweeps, respectively. (**b**–**d**) Schematic configurations illustrating FGaT near positive OOP saturation, the development of a multidomain configuration, and subsequent magnetic reconfiguration during the decreasing-field sweep. The Py arrows illustrate possible responses to the applied field and interfacial magnetic interactions. The arrows in figures (**b**–**d**) indicate the magnetization directions of the magnetic domains.

Compared with the isolated Py film, which exhibits only narrow and nearly symmetric low-field AMR peaks arising from a small angular misalignment, the FGaT/Py heterostructure displays substantially broad multipeak responses. The angular-dependent measurements reveal a clear distinction between the isolated Py film and the FGaT/Py heterostructure. In comparison, the FGaT/Py heterostructure exhibits a substantially broader and more complex multipeak response than that of either constituent device. These features are most pronounced within the field range associated with the OOP magnetization and domain evolution of FGaT, suggesting that interfacial interactions modify the Py magnetization reversal pathway so that it no longer follows the single symmetric pathway observed in the isolated Py device. As FGaT evolves from a nearly saturated state into a labyrinthine multidomain configuration, the domains and associated domain walls produce an interface-associated effective field. Therefore, the Py film experiences different nucleation and depinning conditions, resulting in a distributed, multistep magnetization reversal in the device. The systematic angular evolution therefore suggests that the longitudinal resistance of the heterostructure reflects the coupled response of perpendicular FGaT domains and the Py magnetization. Nevertheless, the transport measurements alone do not identify the magnetic configurations corresponding to the individual resistance features. To elucidate their possible origin, we focus on the OOP magnetoresistance curve of the heterostructure and correlate three representative resistance states with the proposed domain evolution of FGaT and the associated response of the Py magnetization, as illustrated in Figure 4.

Figure 4 presents a possible domain-mediated interfacial pinning scenario for the broadened multipeak resistance response. The three states are therefore schematic representations of the proposed mechanism rather than experimentally resolved configurations of the individual layers. Taking the decreasing-field process as an example, state 1 represents FGaT near positive OOP saturation, with the applied field tending to cant the Py magnetization toward the positive OOP direction. As the field approaches zero, state 2 depicts a possible labyrinthine multidomain configuration of FGaT. The images in Figure 2e,f confirm the presence of such domains in the separate characterization flake. Within the proposed scenario, the FGaT domains and associated domain walls may modify the interfacial magnetic environment, influencing the canting, pinning, and reversal of Py moments. As the negative field increases in magnitude, progressive depinning and magnetic reconfiguration of Py could occur, as illustrated schematically by state 3. The depth-dependent Py magnetization is not resolved by the present measurements. More generally, studies of SrFe_12_O_19_/CoFe_2_O_4_ nano-heterostructures have shown that interfacial exchange coupling can modify the degree of correlated magnetic reversal between components with different anisotropies [36]. This provides a useful framework for understanding the proposed interfacial modulation in FGaT/Py. In this mixed-dimensional structure, the evolving domain configuration of perpendicular FGaT may alter the reversal barriers experienced by the adjacent in-plane soft-ferromagnetic Py layer, providing a possible basis for the broadened resistance response and its dependence on magnetic-field history. In addition, MOKE measurements further corroborate the domain evolution and the formation of labyrinthine domains in FGaT (Figure S4). Under the larger positive field excursion, the Kerr response reaches a well-defined upper plateau before the field is reversed. The corresponding images show changes in the magnetic contrast and domain configuration during the return sweep. The different return-branch profile indicates that the magnetic response depends on the preceding field excursion. This observation is consistent with the proposed role of FGaT domain evolution in establishing field-dependent interfacial pinning conditions and modifying the resistance loops of the heterostructure. The reversal scenario in Figure 4 is inferred mainly from transport data, with magnetic imaging providing supporting context. It is consistent with the multipeak response, hysteresis, and turning-field dependence.

**Figure 5:**
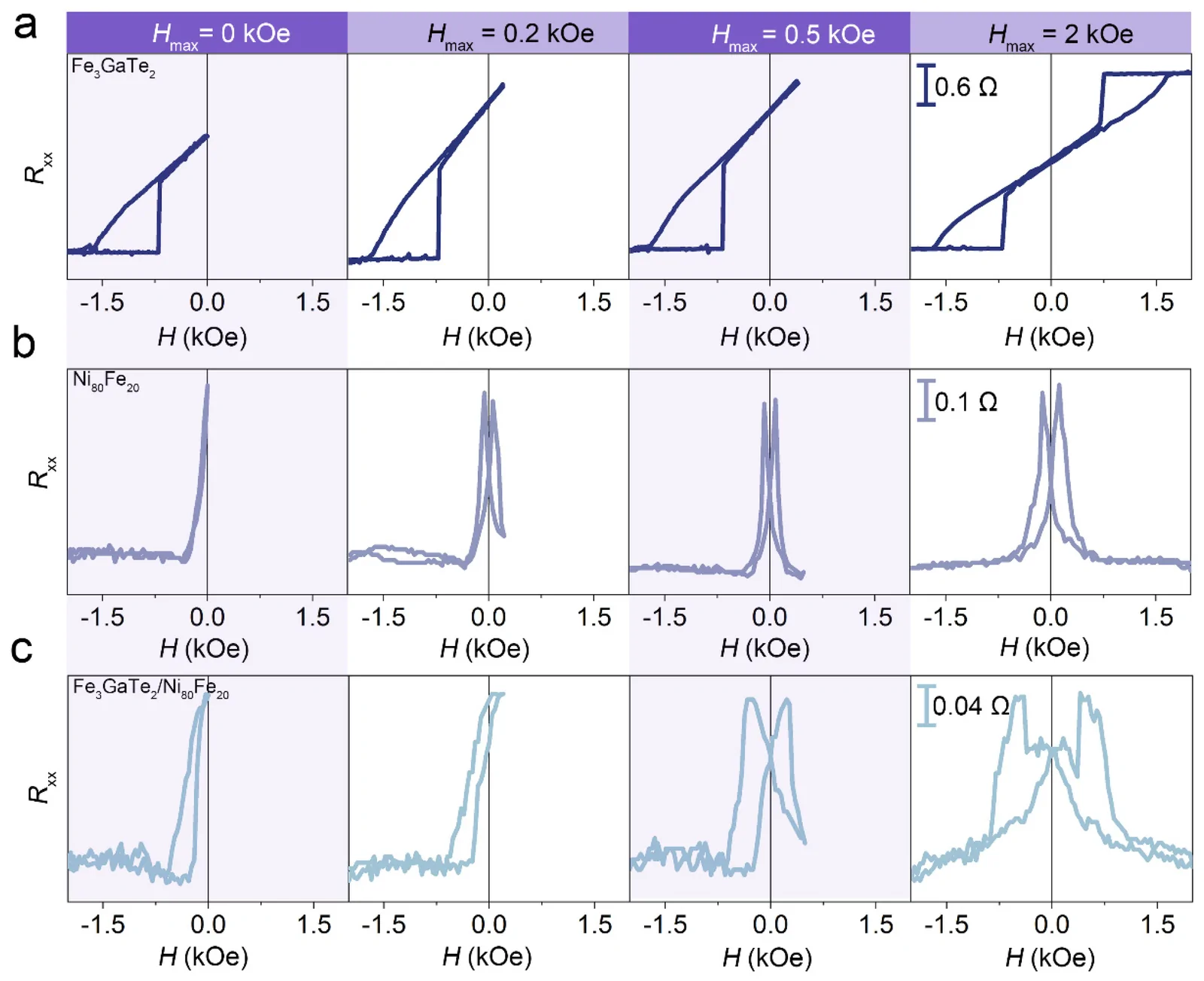
Field-dependent minor-loop magnetoresistance of Fe_3_GaTe_2_, Py, and the Fe_3_GaTe_2_/Py heterostructure. Longitudinal resistance measured using asymmetric field-sweep sequences from −2 kOe to positive reversal fields of 0, 0.2, 0.5, and 2 kOe and subsequently back to −2 kOe. (**a**) Transport curves of Fe_3_GaTe_2_, showing the progressive opening of the minor loops as the positive reversal field is increased. (**b**) Transport curves of Py, showing relatively localized low-field AMR peaks with a weaker dependence on the scan range. (**c**) Transport curves of Fe_3_GaTe_2_/Py heterostructure, exhibiting reversal-field-dependent broadened multipeak responses and pronounced hysteretic effects.

The scenario proposed in Figure 4 motivates an examination of how the preceding field excursion influences the subsequent resistance response. In a multidomain magnet, different field histories may establish different magnetic configurations even at the same applied field. In particular, varying the positive turning field may drive FGaT through different stages of domain evolution, thereby establishing distinct interfacial pinning landscapes and Py configurations. Therefore, we next investigate the sweeping-field-dependent magnetoresistance behavior (Figure 5). To examine the influence of the perpendicular magnetic-field, asymmetric minor-loop measurements were performed using the sequence (−2 kOe ~ *H*_max_ ~ −2 kOe), where (*H*_max_) was set to 0, 0.2, 0.5, and 2 kOe. As shown in Figure 5a, the Fe_3_GaTe_2_ resistance loop progressively opens with increasing (*H*_max_), indicating that the positive reversal field controls the fraction and configuration of the reversed perpendicular magnetic domains. In contrast, the isolated Py film retains a relatively stable low-field response as *H*_max_ is varied. Its resistance features remain confined to a narrow field range and mainly originate from the AMR response associated with the field-induced canting and reversal of the Py moments. This weak dependence on *H*_max_ indicates that the asymmetric field excursion alone is insufficient to generate the broadened multipeak response and pronounced zero-field hysteresis observed in the heterostructure.

Notably, the FGaT/Py heterostructure exhibits a substantially stronger dependence on the turning field *H*_max_. As *H*_max_ is varied, the resistance curve evolves from an incomplete asymmetric loop into a broad multipeak response with pronounced hysteresis. The most pronounced change is observed at *H*_max_ = 0.2 kOe, which lies within the domain-reversal field range of Fe_3_GaTe_2_, suggesting that the zero-field resistance is closely related to the magnetic configuration of FGaT. Within the proposed mechanism, *H*_max_ determines the extent to which the FGaT domains are nucleated, expanded, or driven toward positive saturation. A possible interpretation is that different turning fields establish different remanent FGaT configurations, which may modify the magnetic interactions experienced by Py and contribute to the subsequent resistance response. History-dependent domain formation has been demonstrated in the related van der Waals ferromagnet FGaT [9], providing a relevant context for this interpretation. Importantly, asymmetric resistance minor loops should not be interpreted as evidence of conventional exchange bias. Restricted field excursions can leave magnetic components incompletely reversed, and studies of soft–hard magnetic composites have also demonstrated loop shifts influenced by dipolar interactions and asymmetric reversal [37]. We therefore describe the present observations as field-history-dependent magnetotransport, without assigning the loop asymmetry to exchange-bias mechanism. When the field returns to zero, the remanent FGaT domain configuration may generate a local magnetostatic field in the adjacent Py layer, thereby modifying its magnetic configuration and reversal barriers. This provides a possible explanation for the history-dependent resistance.

The reconstructed domain configuration persists after removal of the field and stabilizes interfacial Py configuration, producing a hysteretic, nonvolatile-type resistance response. Field-resolved MOKE measurements provide additional evidence for magnetic-domain evolution in the heterostructure (Figure S5). The images acquired at the five marked states show changes in the magnetic contrast and domain configuration along the outgoing and return branches of the asymmetric field cycle. These observations support a reversal process involving domain evolution in FGaT, which may modify the interfacial magnetic environment and the pinning conditions experienced by the adjacent Py layer. These results demonstrate that *H*_max_ acts as a control parameter for selecting the remanent magnetotransport state of the FGaT/Py heterostructure. The comparison with the isolated constituents further suggests that this field dependence cannot be explained by the intrinsic response of Py alone but is consistent with domain-mediated interfacial modulation of the Py reversal process.

## 4. Conclusions

In summary, we constructed a mixed-dimensional FGaT/Py heterostructure combining a perpendicular van der Waals ferromagnet with an IP soft magnetic film. Magnetic measurements confirm their magnetic anisotropies, while MFM and MOKE imaging reveals the labyrinthine multidomain configuration of FGaT. Compared with the narrow and nearly symmetric low-field AMR features of isolated Py, the heterostructure exhibits broadened multipeak resistance responses and pronounced hysteresis. The occurrence of these features within the FGaT domain-reversal range supports a domain-mediated interfacial pinning mechanism. In this viewpoint, the formation and evolution of FGaT labyrinthine domains modify the interfacial magnetic environment and pin the Py moments near the interface, thereby altering the depinning conditions and promoting a multistep Py reversal. Moreover, asymmetric minor-loop measurements show that the switching field *H*_max_ controls the extent of FGaT domain reversal and consequently selects different remanent interfacial and Py magnetic configurations. These configurations are electrically detected as distinct zero-field resistance states, including nonhysteretic and hysteretic nonvolatile-type responses. The results establish multidomain van der Waals magnets as active interfacial control layers for reconfiguring the magnetic reversal and remanent magnetotransport of conventional soft-ferromagnetic films.

## Data Availability

The original contributions presented in this study are included in the article and Supplementary Materials. Further inquiries can be directed to the corresponding author.

## Supplementary Materials

The following supporting information can be downloaded at: https://www.mdpi.com/article/10.3390/nano16201304/s1, Figure S1. AFM height profiles of the FGaT flakes used for (a) the FGaT/Py heterostructure device and (b) MFM and MOKE characterization. Figure S2. OOP MOKE hysteresis loop of ultrathin Fe_3_GaTe_2_, showing the Kerr rotation angle (*θ*_K_) as a function of magnetic field. The nearly rectangular loop with abrupt switching is consistent with its perpendicular magnetic easy axis. Figure S3. Hall resistance (*R*_xy_) of an isolated thicker Fe_3_GaTe_2_ device as a function of the OOP magnetic field. The hysteretic anomalous Hall response reflects the reversal of the perpendicular magnetization. Figure S4. Field-dependent MOKE characterization of the Fe_3_GaTe_2_/Py heterostructure. (a) OOP MOKE hysteresis loop with five representative states labeled ①–④. (b–e) Corresponding MOKE images showing the evolution of magnetic contrast and domain configurations during the field cycle. Figure S5. MOKE characterization of the Fe_3_GaTe_2_/Py heterostructure under an asymmetric out-of-plane field cycle. (a) *θ*_K_ as a function of magnetic field, with five representative states labeled (1)–(4). (b–e) Corresponding MOKE images showing the evolution of magnetic contrast and domain configurations along the outgoing and return branches.
