# Supplementary material for: Interface-Induced Magnetization Reversal and Multistate Magnetoresistance in Fe3GaTe2/Ni80Fe20 Heterostructure
Source: Nanomaterials (Basel). 2026 Oct 15;16(20):1304. doi: 10.3390/nano16201304 (PMC13707998; doi:10.3390/nano16201304)
Supplement: Supplementary file 1 [file nanomaterials-16-01304-s001.zip › nanomaterials-4594581-supplementary.pdf]

Support information

Interface-Induced Magnetization Reversal and Multistate Magnetoresistance in $Fe_3GaTe_2/Ni_{80}Fe_{20}$ Heterostructure

Guangcheng Wang[1], Lin Ma[2], Shuo Xu[3, 4], Jihui Li[1], Meiyin Yang[3,4], Zijing Zhao[1*], Xiaolei Wang[1*]

1 School of Physics and Optoelectronic Engineering, Beijing University of Technology, Beijing 100124, China.

2 Key Laboratory of Advanced Functional Materials, Ministry of Education, College of Materials Science and Engineering, Beijing University of Technology, Beijing 100124, China

3 Key Laboratory of Fabrication Technologies for Integrated Circuits, Chinese Academy of Sciences, Beijing 100029, China.

4 Institute of Microelectronics of the Chinese Academy of Sciences, Beijing 100029, China.

* Correspondence: zhaozijing@bjut.edu.cn; xiaoleiwang@bjut.edu.cn

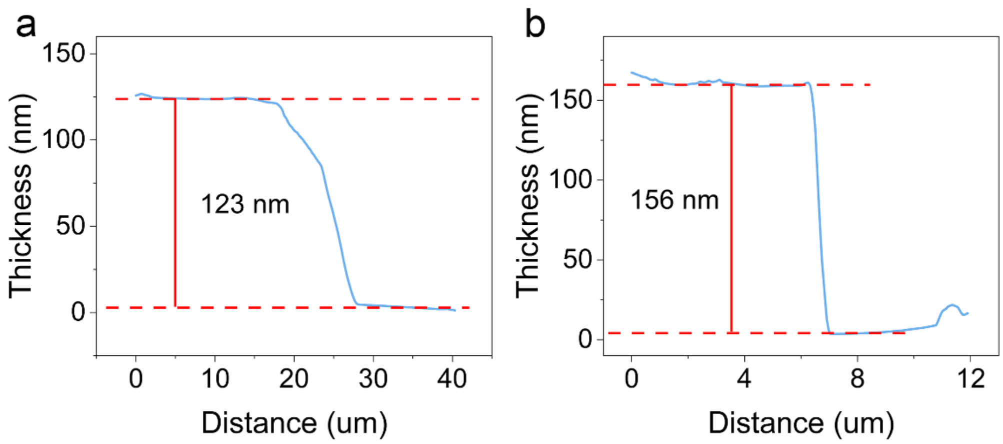

Figure S1. AFM height profiles of the FGaT flakes used for (a) the FGaT/Py heterostructure device and (b) MFM and MOKE characterization.

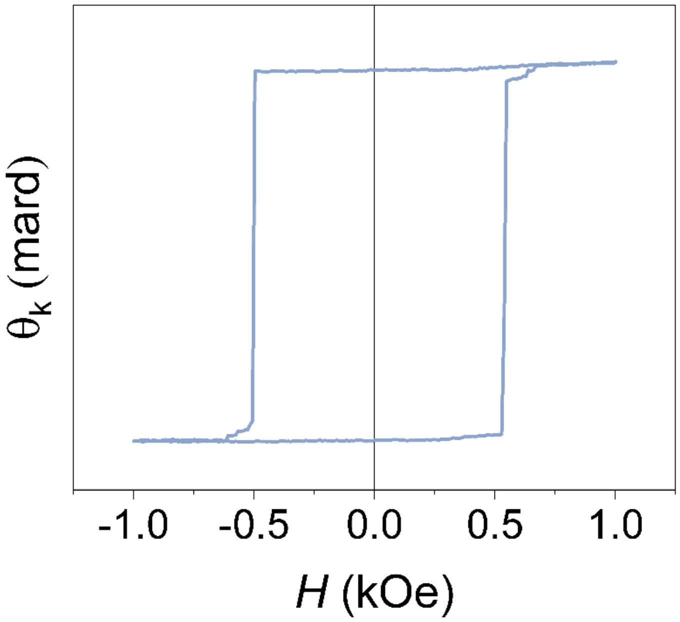

Figure S2. OOP MOKE hysteresis loop of ultrathin Fe_3GaTe_2, showing the Kerr rotation angle (θ_K) as a function of magnetic field. The nearly rectangular loop with abrupt switching is consistent with its perpendicular magnetic easy axis.

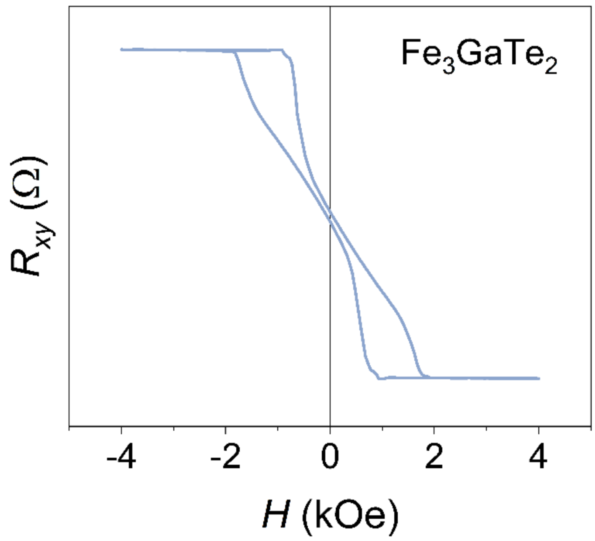

Figure S3. Hall resistance (R_{xy}) of an isolated thicker Fe_3GaTe_2 device as a function of the OOP magnetic field. The hysteretic anomalous Hall response reflects the reversal of the perpendicular magnetization.

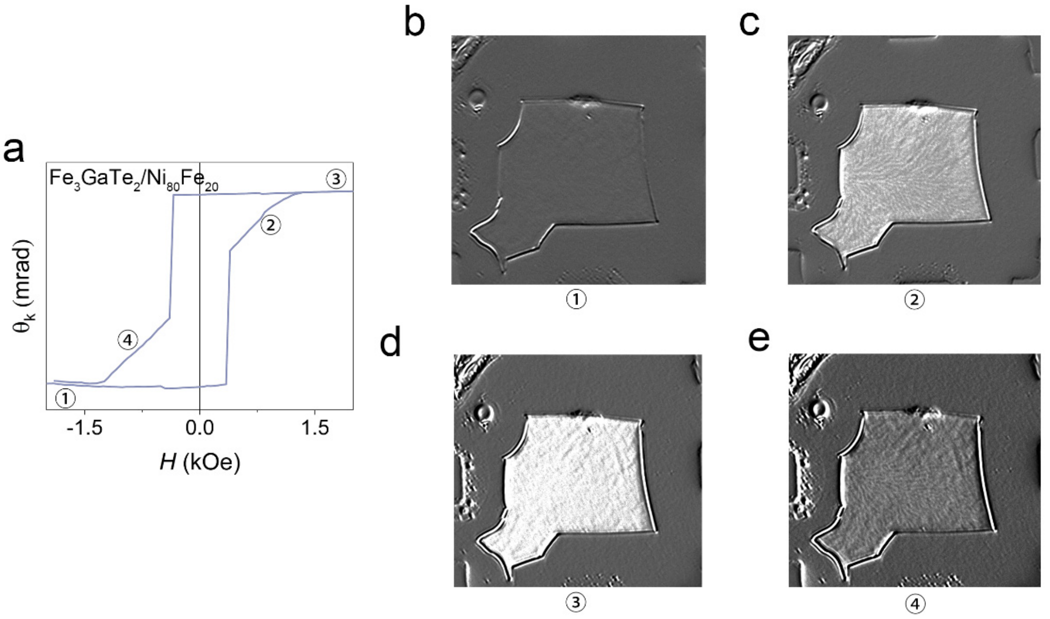

Figure S4. Field-dependent MOKE characterization of the Fe_3GaTe_2/Py heterostructure. (a) OOP MOKE hysteresis loop with five representative states labeled ①–④. (b–e) Corresponding MOKE images showing the evolution of magnetic contrast and domain configurations during the field cycle.

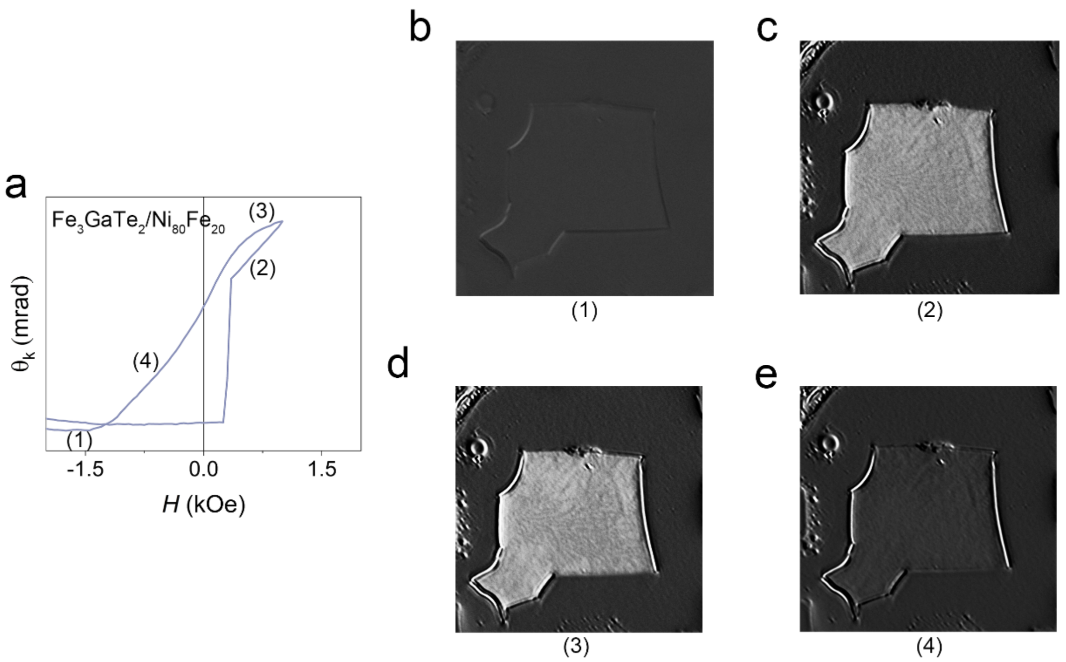

Figure S5. MOKE characterization of the Fe_3GaTe_2/Py heterostructure under an asymmetric out-of-plane field cycle. (a) θ_K as a function of magnetic field, with five representative states labeled (1)–(4). (b–e) Corresponding MOKE images showing the evolution of magnetic contrast and domain configurations along the outgoing and return branches.
